# The effect of focused lung ultrasonography on antibiotic prescribing in patients with acute lower respiratory tract infections in Danish general practice: study protocol for a pragmatic randomized controlled trial (PLUS-FLUS)

**DOI:** 10.1186/s13063-024-08129-2

**Published:** 2024-05-02

**Authors:** Julie Jepsen Strøm, Camilla Aakjær Andersen, Martin Bach Jensen, Janus Laust Thomsen, Christian B. Laursen, Søren Helbo Skaarup, Hans Henrik Lawaetz Schultz, Malene Plejdrup Hansen

**Affiliations:** 1 Center for General Practice at Aalborg University, Aalborg, Denmark; 2 Department of Respiratory Medicine, Odense University Hospital, Odense, Denmark; 3 Institute of Clinical Research, University of Southern Denmark, Odense, Denmark; 4 Department of Respiratory Medicine and Allergy, Aarhus University Hospital, Aarhus, Denmark; 5 Department of Cardiology, Section for Lung Transplantation, Rigshospitalet, Copenhagen, Denmark

**Keywords:** Community-acquired pneumonia, Acute lower respiratory tract infections, Focused lung ultrasonography, Antibiotics, Adults, General practice, Randomized controlled trial

## Abstract

**Background:**

The use of antibiotics is a key driver of antimicrobial resistance and is considered a major threat to global health. In Denmark, approximately 75% of antibiotic prescriptions are issued in general practice, with acute lower respiratory tract infections (LRTIs) being one of the most common indications. Adults who present to general practice with symptoms of acute LRTI often suffer from self-limiting viral infections. However, some patients have bacterial community-acquired pneumonia (CAP), a potential life-threatening infection, that requires immediate antibiotic treatment. Importantly, no single symptom or specific point-of-care test can be used to discriminate the various diagnoses, and diagnostic uncertainty often leads to (over)use of antibiotics. At present, general practitioners (GPs) lack tools to better identify those patients who will benefit from antibiotic treatment. The primary aim of the PLUS-FLUS trial is to determine whether adults who present with symptoms of an acute LRTI in general practice and who have FLUS performed in addition to usual care are treated less frequently with antibiotics than those who only receive usual care.

**Methods:**

Adults (≥ 18 years) presenting to general practice with acute cough (< 21 days) and at least one other symptom of acute LRTI, where the GP suspects a bacterial CAP, will be invited to participate in this pragmatic randomized controlled trial. All participants will receive usual care. Subsequently, participants will be randomized to either the control group (usual care) or to an additional focused lung ultrasonography performed by the GP (+ FLUS). The *primary outcome* is the proportion of participants with antibiotics prescribed at the index consultation (day 0). Secondary outcomes include comparisons of the clinical course for participants in groups.

**Discussion:**

We will examine whether adults who present with symptoms of acute LRTI in general practice, who have FLUS performed in addition to usual care, have antibiotics prescribed less frequently than those given usual care alone. It is highly important that a possible reduction in antibiotic prescriptions does not compromise patients’ recovery or clinical course, which we will assess closely.

**Trial registration:**

ClinicalTrials.gov NCT06210282. Registered on January 17, 2024.

## Administrative information

Note: The numbers in curly brackets in this protocol refer to SPIRIT checklist item numbers. The order of the items has been modified to group similar items (see http://www.equator-network.org/reporting-guidelines/spirit-2013-statement-defining-standard-protocol-items-for-clinical-trials/).

**Table:** 

Title {1}	The effect of focused lung ultrasonography on antibiotic prescribing in patients with acute lower respiratory tract infections in Danish general practice: Study protocol for a pragmatic randomized controlled trial (PLUS-FLUS)
Trial registration {2a and 2b}.	ClinicalTrials.gov. Identifier number: NCT06210282.
Protocol version {3}	Version 4.0. 01.11.2023.
Funding {4}	The PLUS-FLUS Trial is financially supported by: The Health Foundation (20-B-0048), The Foundation for General Practice (A4162), The Lung Associations’ Research Fund (A73) and The Quality and Education’s committees for General Practice in the Region of Southern Denmark, the Capital Region, and the Region of Northern Jutland.
Author details {5a}	Julie Jepsen Strøm^1^ (JJS), Primary Investigator (PI) Camilla Aakjær Andersen^1^ (CAA) Martin Bach Jensen^1^ (MBJ) Janus Laust Thomsen^1^ (JLT) Christian B. Laursen^2,3^ (CBL) Søren Helbo Skaarup^4^ (SHS) Hans Henrik Lawaetz Schultz^5^ (HHS) Malene Plejdrup Hansen^1^ (MPH) ^1^ Center for General Practice at Aalborg University, Aalborg, Denmark. ^2^ Department of Respiratory Medicine, Odense University Hospital, Odense, Denmark. ^3^ Institute of Clinical Research, University of Southern Denmark, Odense, Denmark. ^4^ Department of Respiratory Medicine and Allergy Aarhus University Hospital, Aarhus, Denmark. ^5^ Department of Cardiology, Section for lung transplantation, Rigshospitalet, Copenhagen, Denmark
Name and contact information for the trial sponsor {5b}	Center for General Practice at Aalborg University (CAM AAU), Department of Clinical Medicine, Aalborg University. Selma Lagerløfs Vej 249, 11.02.043 DK-9260 Gistrup Denmark
Role of sponsor {5c}	Sponsor is noncommercial and declares no conflict of interest. Sponsor and funders had no role in the study design and will have no role in conducting the study, analysing the results, writing the report, nor in the descision to submit the report for publication.

## Introduction

### Background and rationale {6a}

The World Health Organization (WHO) states that antimicrobial resistance is one of the largest threats to global health [1]. Antibiotic use is the main driver of the selection of resistant bacteria [2], and resistance develops quickly after any use of antibiotics. Ninety percent of all antibiotic prescriptions in Denmark are prescribed in primary care [3]. General practitioners (GPs) issue approximately 75% of these prescriptions, with acute lower respiratory tract infections (LRTIs) being one of the most common indications [4].

The term ‘acute LRTI’ includes several different conditions—with overlapping symptoms—for example, acute bronchitis caused by viruses or bacterial community-acquired pneumonia (CAP) [5]. Importantly, no single symptom or specific C-reactive protein (CRP) cut-off value can be used to discriminate the various diagnoses [6, 7]. It is well known that this diagnostic uncertainty often leads to (over)use of antibiotics [8, 9], and there is a large need for developing and testing new tools in the general practice setting to help differentiate between benign self-limiting acute LRTIs and bacterial CAPs.

Several studies have demonstrated that focused lung ultrasound (FLUS) has excellent accuracy for diagnosing pneumonia in hospitalized adults [10–15]. However, FLUS is not commonly used by GPs [16], even though GPs are increasingly using point-of-care ultrasonography (POCUS) for a wide range of purposes [17]. To date, the only study in which FLUS was used to guide antibiotic prescription in patients with acute LRTIs, performed in a general practice setting, was a randomized trial by L’Hoptallier et al. [18]. The intervention consisted of a sequential procalcitonin point-of-care test and FLUS combined with usual care, where FLUS was only applied if a specific procalcitonin cut-off value was reached. Only nine patients had FLUS performed, and no additive effect of FLUS on antibiotic prescribing was shown. Consequently, there is a need for a trial determining whether FLUS alone, as an addition to usual care, has the potential to reduce antibiotic prescribing in patients presenting to general practice with symptoms of an acute LRTI.

### Objectives {7}

The primary aim of the PLUS-FLUS Trial is to determine if adults presenting with symptoms of an acute LRTI in general practice where the GP suspects CAP, who have FLUS performed as an addition to usual care, have antibiotics prescribed less frequently than those given usual care.

We hypothesize that FLUS, added to usual care, will lead to a significant decrease in the proportion of participants who have antibiotics prescribed.

Moreover, this trial aims to compare the clinical course of participants in the intervention group and control group in terms of the duration and burden of LRTI symptoms through a patient-reported LRTI symptom diary at days 0–21 as well as antibiotics prescribed after index consultation up to day 28, number of reconsultations, imaging performed, illness deterioration (hospitalization, complications, all-cause mortality), referral for suspicion of cancer, number of cancer diagnoses, and unintended events detected in a review of medical records from days 0 to 60. Additionally, participants’ satisfaction with the index consultation will be compared between groups.

### Trial design {8}

This is the protocol for a pragmatic, randomized superiority trial with a two-group parallel design. The unit of randomization is the patient, and the allocation ratio between the control and intervention groups is 1:1. This protocol follows the Standard Protocol Items: Recommendations for Interventional Trials, 2013 statement [19].

## Methods: participants, interventions, and outcomes

### Study setting {9}

Adults with acute cough (< 21 days) and at least one other symptom of acute LRTI, where the GP suspects a bacterial CAP, will be recruited by expected 30–40 GPs distributed in general practice clinics in all five geographical regions of Denmark. GPs in Denmark are self-employed and work in office-based general practice clinics. There are 3.488 GPs in Denmark distributed in approximately 1700 general practice clinics, as more than 60% of clinics have two or more GPs associated [20]. Almost all inhabitants in Denmark are listed with a GP [21], and GPs act as gatekeepers for other primary care healthcare providers and secondary care specialists. Patients are free of charge for consultations, as GPs are paid through a combination of remuneration and fee-for-service financed through taxes. However, there is no fee for performing POCUS in general practice, and GPs must cover expenses for the ultrasound device and their ultrasound education themselves. Participating GPs in the PLUS-FLUS Trial will be economically compensated for the time they spend on recruitment and data collection, including performing FLUS. The standard payment recommended by the Danish College of General Practitioners (146,37 DKK ≈ 20 EUR/10 min) will be used.

### Eligibility criteria {10}

To be eligible for the study, patients must fulfil all the inclusion criteria:

1. Age ≥ 18 years
2. Acute cough (< 21 days)
3. At least one other symptom of LRTI
4. The GP suspects a bacterial CAP

The presence of any of the following exclusion criteria leads to patient exclusion from the study:

1. Previous antibiotic treatment for the current episode of acute LRTI
2. The patient is not listed with the GP (no medical record available)
3. The patient is not capable of understanding or signing informed consent
4. The patient does not wish to participate in the study

The GPs will be recruited through our broad GP network, social media for GPs using POCUS in general practice, and collaboration with the Danish Society for Ultrasonography in General Practice. If more GPs sign up for participation than the capacity of the project holds, participating GPs will be selected to constitute a population as diverse as possible based on demographics, experience with POCUS, experience with FLUS, organizational aspects of the clinic, and experience as a general practitioner.

To be eligible for the study, GPs who will perform the interventions must fulfil all the following criteria:

1. A specialist in general medicine
2. POCUS (but not necessarily FLUS) should be used at least once a week in general practice or as an off-hours service
3. Prior to the study period and recruitment of patients, have participated in a FLUS training program with a validated assessment

### Who will take informed consent? {26a}

Eligible participants will be informed about the PLUS-FLUS Trial and provided with an information leaflet, an informative sheet titled ‘The Rights of a Trial Subject in a Health Scientific Research Project’, and a consent form from the GP. The GP will ensure that the patient understands the purpose of the project, potential benefits, risks, and procedures involved. The GP will underscore that participation is voluntary and that the patient can decline to participate or withdraw from the project at any time without consequences. The patient is informed that consent to participate will give the primary investigator (PI), sponsor, and controlling authorities access to obtain information in the patient’s medical records, including the electronic records and the patient’s shared medication records (i.e. FMK), to obtain information about the patient’s health conditions.

Due to the nature of the project, the patient will only be given a few minutes to consider participation. The GP will check patients against the eligibility criteria stated above and invite patients to participate if they fulfil all the inclusion criteria and none of the exclusion criteria. Patients who agree to participate will be asked to provide written consent, which will be obtained by the GP.

### Additional consent provisions for collection and use of participant data and biological specimens {26b}

Informed consent is obtained prior to the collection of participant data. Participants are informed about the storage and use of their data, including subsequent merging with data from the national registries.

## Interventions

### Explanation for the choice of comparators {6b}

Participants assigned to the control group will receive the GP’s usual care of adults (≥ 18 years) presenting with symptoms of an acute LRTI where the GP suspects CAP. Usual care will be used as a pragmatic comparator to reflect the current standard examinations and care of these patients in general practices in Denmark. Usual care is recommended to follow applicable guidelines from the Danish Society of General Practitioners (DSAM) [22] and Lægehåndbogen [The Doctor’s Handbook] [23]. FLUS is currently not a standard or even a common examination in Danish general practice. Even for GPs already using POCUS on a weekly basis, FLUS is not part of usual care for adults presenting with symptoms of an acute LRTI.

### Intervention description {11a}

Participants assigned to the intervention group will receive a FLUS examination during the index consultation (day 0) in addition to usual care.

#### Ultrasonography equipment

The participating GPs use POCUS on a weekly basis before trial commencement and use the ultrasonography device already available to them. The type of device (classified as hand-held, laptop, or stationary), brand, model, and transducer used will be reported.

#### FLUS training program

A pilot test of a FLUS training programme for GPs was performed in a prospective cohort study (ClinicalTrials.gov NCT04711031), after which the programme was adjusted to fit this trial. GPs who have not already participated in a FLUS training program with a validated assessment will complete this FLUS training program before trial commencement.

The FLUS training program consists of theoretical self-studying, a 1-day hands-on FLUS training course, and, subsequently, specialist-supervised scans. The theoretical part of the training programme consists of an estimated four-hour self-study based on e-learning material from *Basal Klinisk Ultralydsdiagnostik* [Basic Clinical Ultrasound Diagnostics] published by Munksgaard [24]. The self-study concludes with a validated theoretical multiple-choice question test with a pass/fail assessment [25], which must be passed prior to the FLUS hands-on training course.

The 1-day FLUS training course consists of an introduction followed by five hours of hands-on training on (1) healthy persons and (2) a simulator. The simulator resembles an ultrasound device and combines a mannequin torso with dynamic images when scanned. At the end of the day, each GP completes a simulation-based test, and a Lung Ultrasound-Objective Structured Assessment of Ultrasound Skills (LUS-OSAUS) score will be obtained for FLUS skills [26].

As part of the training programme, each GP is encouraged to perform five to ten FLUS examinations in their clinic within 1 month following the training course. FLUS specialists from Danish university hospitals (Department of Respiratory Medicine at Odense and Aarhus University Hospitals and Department of Cardiology, Section for Lung Transplantation, Rigshospitalet, Copenhagen University Hospital) supervise the GPs on the ultrasound images obtained from the examinations and their interpretation of findings. The LUS-OSAUS scores and number of supervised FLUS examinations will be reported for participating GPs.

#### 14-zone FLUS scanning protocol

The FLUS scanning protocol used has been previously validated in hospital settings [27, 28] and recently also in a general practice setting [29]. Moreover, before trial commencement, the FLUS scanning protocol was pilot tested in a similar patient population in a prospective cohort study in general practice (ClinicalTrials.gov NCT04711031). The hemithorax is divided into anterior, lateral, and posterior surfaces. The anterior and lateral surfaces on each hemithorax are divided into upper and lower quadrants. The posterior surfaces on each hemithorax are divided into upper, middle, and lower quadrants. Each quadrant represents a scanning zone. The scanning zones on the patients’ left side can be denoted from 1 to 7L, and those on the right side can be denoted from 1 to 7R. Each scanning zone is assessed using FLUS. The transducer is placed in the middle of the scanning zone to create a cross-sectional image of the intercostal space and the underlying pleura blades. The positioning of the patient during the examination is decided by the GP and can be in an upright or a supine position or both. The GPs are trained in the 14-zone scanning approach during the FLUS training program.

#### FLUS pathological findings

FLUS pathological findings are predefined, and the GPs are trained in recognizing and defining possible pathological findings during the FLUS training program. The definitions of FLUS findings are based on the European Federation of Societies for Ultrasound in Medicine and Biology (EFSUMB) coursebook [30] and findings described in a similar patient population through a prospective cohort study in general practice (ClinicalTrials.gov NCT04711031).

- B-lines: Laser-like vertical echogenic artefacts arising from the pleural line, spreading without fading to the edge of the screen and moving synchronously with lung sliding
- Interstitial syndrome: Multiple (≥ 3) B-lines in at least 2 zones on each side present
- Consolidation: Loss of aeration, which allows visualization of the lung parenchyma with sonomorphologic characteristics that resemble a solid organ or tissue. Pathognomonic for a pneumonic consolidation is the presence of air-bronchograms and serrated or blurred margins
- Subpleural consolidation: Small subpleural consolidation between 2 and 20 mm in size that moves together with lung sliding
- Pleural effusion: Anechoic or hypoechoic space between the visceral and parietal pleura
- Focal visceral pleural pathology: Hypoechogenic thickening of the pleura with a rough appearance and interruption of the normally smooth pleura
- Pneumothorax: Area without lung sliding, lung pulse or B-lines, with the presence of a lung point in an adjacent area
- Other FLUS pathology: Other incidental findings by FLUS are described according to the ability of the GPs

### Criteria for discontinuing or modifying allocated interventions {11b}

Due to the short intervention period during index consultation, the allocated intervention may only be discontinued for a given trial participant by the GP due to an unexpected event, hindering the GP from conducting or completing the allocated intervention, e.g. acute worsening of the participant’s condition during index consultation, after consent has been given and allocation has been revealed. Moreover, the study may be discontinued for a given trial participant upon participant request or by withdrawal of informed consent. The data collected before the discontinuation or withdrawal of consent will be retained and used in the analyses; however, no further data will be obtained from the participant.

### Strategies to improve adherence to interventions {11c}

During the trial, participating GPs will be contacted by the PI at least twice a month to improve adherence to the intervention protocols.

### Relevant concomitant care permitted or prohibited during the trial {11d}

Participants assigned to the control group are prohibited from having a FLUS performed by the GP during the index consultation. No further concomitant care or interventions are prohibited during the index consultation or during the follow-up period.

### Provisions for post-trial care {30}

The participating patients are covered by the Danish Patient Compensation Association, as the consultations are performed by GPs (authorized health care professionals). Participating GPs are covered by the Occupational Injuries Insurance Act [31].

### Outcomes {12}

#### Primary outcome

The primary outcome is the proportion of participants with antibiotics prescribed at the index consultation (day 0) reported by the GP at the index consultation. We will assess the effect of adding FLUS to usual care on antibiotics prescribed at the index consultation by investigating whether there is a difference between groups in the proportion of participants with antibiotics prescribed.

#### Secondary outcome

1. Outcomes from the LRTI symptom diary
  Participants will be asked to complete a validated LRTI symptom diary every day from day 0 to day 21 [32]. The recorded items include the following six symptoms of LRTI: cough, dyspnoea, sputum production, well-being, sleep disturbance, and activity disturbance. The participants are asked to consider how bad each symptom has been over the past 24 h by scoring each symptom on a 7-point Likert scale (0 = no problem, 1 = very little problem, 2 = slight problem, 3 = moderate problem, 4 = bad problem, 5 = very bad problem, and 6 = as bad as it could be). Moreover, the diary contains a social domain on cancellation of work-related or leisure activities [33]. Only on the day of the index consultation (day 0) will the diary also include a question on participants’ satisfaction with the consultation, assessed on a 5-point Likert scale (very dissatisfied (1) to very satisfied (5)) [34]
  1.1) Daily total LRTI symptom score, calculated as the sum of the scores for six symptoms (minimum 0 - maximum 36) (mean/median)
  1.2) The number of days with symptoms rated as ‘moderate problem’ or worse by the participant (at least one item with a score of 3 or above) (mean/median)
  1.3) Number of days participants signed in sick/cancelled work-related activities or cancelled leisure activities(mean/median)
  1.4) Proportion of participants who were satisfied or very satisfied (4 or 5) with the index consultation
  Outcomes 1.1, 1.2, and 1.3 from the LRTI symptom diary will be calculated for each participant every day from day 0 until the participant has scored 0 for every item, whichever comes first, or up to a maximum of 21 days [32].
2. Outcomes from participants’ shared medication records (i.e. FMK) and on type of prescription
  As a standard care and communication method, data on changes in medicine or new prescriptions are automatically uploaded to the participants’ electronic shared medication record (FMK). We will review the participants’ shared medication records (FMK) for outcomes on antibiotics prescribed during follow-up in both groups:
  2.1) Proportion of participants with antibiotics prescribed within 7 days after the index consultation
  2.2) Proportion of participants with antibiotics prescribed within 28 days after the index consultation
  If antibiotics are prescribed at the index consultation (day 0), the GP is asked to report whether the prescription is an immediate or delayed prescription (ref.).
  2.3) Proportion of antibiotics prescribed as delayed antibiotic prescriptions at index consultation (day 0) [35, 36]
  As a standard care and communication, GPs receive notices of health-related events, e.g. discharge and out-of-hour notices. From participants’ electronic medical records, we will obtain outcomes on the clinical course during follow-up in both groups:
3. Outcomes from participants’ electronic medical records
  3.1) Proportion of participants with reconsultations, defined as any primary care contact (general practice or out-of-hour services), within 28 days after the index consultation
  3.2) Proportion of participants admitted to the hospital within 28 days after the index consultation
  3.3)  Proportion of participants with complications (pleural infection (defined as complicated parapneumonic effusion or empyema), lung abscess, or sepsis) during admission to the hospital within 28 days after the index consultation
  3.4) Proportion of participants with imaging other than FLUS (any imaging performed in secondary health care services) performed within 28 days after the index consultation
  3.5) Other imaging methods performed within 28 days after the index consultation
  3.6) Proportion of participants referred with suspicion of cancer within 60 days after the index consultation
  3.7) Proportion of participants diagnosed with cancer within 60 days after the index consultation
  3.8) Number of spontaneously reported unintended events up to 60 days after the index consultation
  3.9) All-cause mortality up until day 28 and day 60

### Participant timeline {13}

**Fig. 1:**
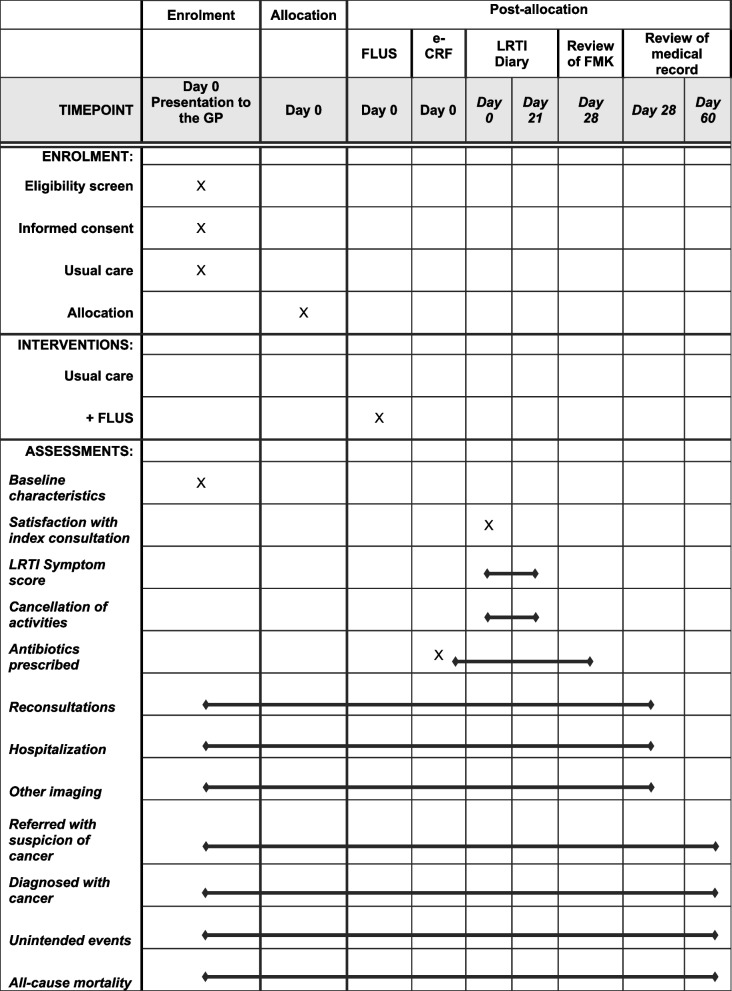
Schedule of enrolment, interventions and assessment. Abbreviations: GP, general practitioner; FLUS, focused lung ultrasonography; e-CRF, electronic case report form; LRTI, lower respiratory tract infection; FMK, shared medication record

A diagram of the enrollment, allocation, intervention, and follow-up of the participants is shown in Fig. 1. Eligible participants are identified as they present to general practice. Once the GP identifies the patient as eligible for the study, information is provided, and informed consent will be signed as described earlier. Following consent, the GP will decide what diagnostics are necessary following the GP’s usual care of patients suggestible of having a bacterial CAP. The GP will complete an electronic case report form (e-CRF) for each participant recording information about symptoms and clinical findings and, if performed, point-of-care test (POCT) results, e.g. CRP. Randomization occurs once usual care has been performed and before possible treatment is prescribed. The GP will proceed directly to the phase of the consultation containing explanation and planning, with participants randomized to the control group before ending the index consultation. Participants randomized to the intervention group will receive a FLUS performed and interpreted by the GP before proceeding to explanation, planning, and ending the consultation. After the index consultation, during follow-up, it is of the discretion of the GP to reassess participants at any time if needed.

### Sample size {14}

Based on previous quality improvement projects in general practice in Denmark [37], a Danish study by Holm et al. [8] and a Dutch study on reducing antibiotic prescribing in patients with LRTI [34], we assume to detect a 15% decrease in antibiotic prescribing in patients with LRTI from 50% (usual care) to 35% (+ FLUS). According to sample size calculations comparing these two proportions, a total of 340 patients with 170 trial participants in each arm were needed, using a 5% significance level and a power of 80%. We assume withdrawal or discontinuation by a maximum of 10% [38], furthermore, we increased the sample size by 5% to account for covariates in the analyses. Consequently, we plan to include a total of 390 trial participants (195 in each arm). Each GP is encouraged to include a minimum of 10 participants to account for the individual effect of FLUS on antibiotic prescribing at a GP level.

### Recruitment {15}

A total of 390 eligible patients will be enrolled by 30–40 GPs. A previous Danish study indicated that the incidence of patients suspected of having CAP when presenting to the GP is three to four patients on average per GP per month during the winter season [39]. Consequently, a study period of five months is expected to include 390 participants. The first participant was enrolled on November 3, 2023, and the enrolment period is envisaged to last until March 31, 2024. Enrollment is monitored by the PI, who contacts participating GPs at least twice a month to maintain recruitment and enrolment.

## Assignment of interventions: allocation

### Sequence generation {16a}

Thirty-six allocation sequences were generated using www.sealedenvelope.com [38]. Permuted block randomization was used to ensure similar enrolment in both groups. Different block sizes were used to prevent the allocation sequence from being anticipated by the GPs. Details on block sizes and list lengths are available in a separate document unavailable to GPs who enroll patients and assign interventions.

### Concealment mechanism {16b}

Thirty-six corresponding piles of sequentially numbered opaque sealed envelopes (SNOSEs) [38] were prepared from the allocation sequences. The allocation sequences and SNOSE piles are marked with corresponding numbers. The allocation sequences are saved on a secure server at Aalborg University that members from the research group do not have access to. Each GP has been provided with a pile of SNOSEs prepared from one allocation sequence. At the time of randomization, the GP will draw the top SNOSE in which group allocation is revealed.

### Implementation {16c}

The allocation sequences and corresponding SNOSEs were prepared by two remote independent researchers who are available for generating more allocation sequences and SNOSE piles if more than 36 GPs participate. The GPs will enroll participants and assign them to either the control or intervention group based on group allocation revealed in the SNOSE.

## Assignment of interventions: blinding

### Who will be blinded {17a}

Owing to the type of intervention, participants and GPs are not blinded to group allocation. The PI and members of the research team involved in obtaining and/or analysing the data (outcome assessors and data analysts) are blinded to group allocation until the data analyses are finalized.

### Procedure for unblinding if needed {17b}

A group of researchers will be overseeing the trial to receive and handle spontaneously reported unintended events from the participating GPs. In each case, the remote group of researchers will assess whether unblinding is necessary. If needed, the PI will contact the remote independent researchers involved in preparing the allocation sequences and provide them with the GP’s SNOSE pile number and the envelope number corresponding to the participant of relevance. The remote independent researchers will gain access to the allocation sequence corresponding to the SNOSE pile number and reveal the allocation group of the participant of relevance.

## Data collection and management

### Plans for assessment and collection of outcomes {18a}

All the data is collected via e-CRFs and surveys through Research Electronic Data Capture (REDCap©), which is a secure web platform for building and managing online databases and surveys.

#### Collection of baseline characteristics of GPs

The baseline characteristics of the participating GPs are collected through email correspondence and registered in REDCap© by the PI. The following information was obtained: age, sex, region of Denmark, type of clinic, seniority as a GP, experience with POCUS, experience with FLUS, type of ultrasound device (hand-held, laptop or stationary), ultrasound brand and model, transducers used, and baseline antibiotic prescribing.

#### Collection of participant data at index consultation (day 0)

The GP will complete an e-CRF on each participant immediately after the index consultation to obtain data on the primary outcome: if antibiotics was prescribed at the index consultation (yes/no).

If an antibiotic was prescribed, the GP is asked about the type of prescription (immediate or delayed).

Moreover, the GP is asked to provide the following information about the participant in the e-CRF:

- Participant baseline characteristics: Date of enrolment, social security number (duplicate registration) from which age and sex were provided, mobile phone number, comorbidities, and smoking status
- Usual care: Participants’ symptoms and signs of acute LRTI, the results of physical examination and any POCT performed as part of usual care (e.g. CRP) (prespecified interval suggested), and participant randomization (SNOSE) number
- FLUS pathological findings (in participants assigned to the + FLUS group)

#### Collection of outcomes from the LRTI symptom diary

The items on LRTI symptoms in the LRTI symptom diary have been validated for use in a randomized controlled trial on the management of acute LRTIs in primary care [32] and prior to the PLUS-FLUS Trial translated into Danish following the Guidelines for the Process of Cross-Cultural Adaptation of Self-reported Measures [40]. The diary for use in acute LRTIs by Watson et al. correlated significantly with the Measure Yourself Medical Outcome Profile 2 (MYMOP2) [41], as did the interpersonal change over time. The standardized response mean, i.e. the mean change in scores divided by the standard deviation (SD) of the diary scores, was 1.48 (> 1), indicating sensitivity to change. Single items from the social domain of the ‘Acute Respiratory Tract Questionnaire’ [33] were also added to the LRTI symptom diary. The diary for the PLUS-FLUS trial was successively pilot tested in a general practice patient population to verify acceptance and face validity.

Data from the LRTI symptom diary will be collected directly into REDCap© through a survey link. Participants will receive the link to the diary every day from day 0 to day 21 through short message services (SMS). REDCap© will be set up to automatically send the survey link as a mail-to-SMS to the participant’s mobile phone number provided in the e-CRF by the GP at the index consultation. The SMSs are sent through an SMS gateway, SureSMS. Participants who do not have a smartphone to receive and open links through SMS, who are not able to receive the diary through SMS for other reasons, or who do not wish to, will be given a paper format diary by the GP. Participants with a paper format diary will be instructed on how to fill in the diary through enclosed written information and are instructed to hand in the paper format diary at the GP’s office after fulfilment. The data from the received paper format diaries will be double entered into REDCap© by the PI, with discrepancies resolved by checking the original data.

#### Collection of outcomes from participants’ shared medication records (i.e. FMK)

Participants’ shared medication records will be evaluated at each general practice. A staff member (e.g. a secretary, nurse or GP in general practice) from a specific general practice will conduct the data extraction from FMK with the PI present to obtain data on antibiotics prescribed up until days 7 and 28. In Denmark, doctors are asked to provide an indication (e.g. pneumonia) for each antibiotic issued. However, members of our research team have previously shown that approximately 1/3 of antibiotic prescriptions issued in Danish general practice are not labelled with an indication [4]. Consequently, our team decided to include any antibiotics prescribed for systemic use (Anatomical Therapeutic Chemical Classification System [ATC] code J01) in the analyses, no matter the indication provided.

#### Collection of outcomes from participants’ electronic medical records

Medical records will be collected from the participating general practices by the PI or a member of the research team. The medical records will be obtained in either paper format or electronically through a portable hard drive and immediately transferred to a secure server at Aalborg University. The medical records must include all journal notes and laboratory and other test results from both primary and secondary care from the index consultation (day 0) up to day 60. First, the medical records will be reviewed and pseudonymized by a researcher who is not part of the research team and who will conceal all the data on or referring to the allocation group. A data extraction tool will be developed in REDCap© and pilot tested. The PI and one other member of the research team will independently review the participants’ medical records and extract the data. We will compare the data obtained, and any disagreements will be resolved by consensus and by consulting the participants’ electronic medical records. If this does not lead to agreement, a third member of the research team will be involved in the final assessment. Data collection forms from the PLUS-FLUS Trial can be obtained from the PI.

### Plans to promote participant retention and complete follow-up {18b}

The loss of follow-up data obtained from medical records and shared medication records is expected to be minimal. Outcome data from medical records and shared medication records will be collected for all participants, except those who are discontinued by the GP or who have withdrawn informed consent before day 60. We expect some participants to be lost to follow-up in the LRTI symptom diary, as we anticipate that some participants will fail to complete the electronic diary or will fail to submit the paper version to the GP. During follow-up, personal daily SMSs will provide participants with an electronic diary. The PI will conduct a standardized phone interview on day 7 with participants filling in a paper format diary and with participants failing to fill in the electronic diary two or more days up until day 7. Date, social security number, and all items of the LRTI symptom diary will be recorded. This approach has been decided on to obtain complete outcome data based on the LRTI symptom diary of day 7 and to motivate participants to complete follow-up.

## Data management {19}

### Data forms and data entry

The data will be collected via e-CRFs and surveys via REDCap©. The e-CRFs and surveys will include range checks for data value, and data on the primary outcome (antibiotic prescribing at the index consultation) are mandatory.

### Data transmission and editing

All user activity in REDCap© is tracked by a built-in audit trail. The data will be transferred directly from REDCap© to the statistical software Stata Version 17, where the data will be processed and analysed. In Stata, there will be an audit trail in the form of a DO-file to document, first, the process of validating the data and how missing data are handled, and subsequently, the analyses will be performed.

### Security and back-up of data

The data are stored and handled in accordance with the stipulations of The Danish Personal Data Protection Act and The General Data Protection Regulation (GDPR). Study data will be stored on a secure server at Aalborg University, and only data processors will have access to the data. Participating GPs handle the data on behalf of the data processors and signed a data management agreement prior to the collection of the data. A data management agreement has also been signed between SureSMS and CAM AAU.

### Confidentiality {27}

Personal information about participants who consent to participate will be stored on a secure server at Aalborg University. All the data will be stored for 10 years after the completion of the study, in accordance with the European Code of Conduct for Research Integrity.

### Plans for collection, laboratory evaluation, and storage of biological specimens for genetic or molecular analysis in this trial/future use {33}

Not applicable. No biological specimens for genetic or molecular analysis will be collected in this trial.

## Statistical methods

### Statistical methods for primary and secondary outcomes {20a}

A detailed description of all analyses will be provided in the Statistical Analysis Plan (SAP) before the end of participant enrolment. All the statistical analyses of the trial are considered a priori analyses. If any post hoc analyses are conducted, they will be defined as such in the report.

The primary outcome data will be displayed in a 2 × 2 table comparing the dichotomous outcome variable of antibiotics prescribed at the index consultation. The primary analyses will be the proportion of patients prescribed an antibiotic at the index consultation (day 0) in the two groups. The primary analyses will also include the risk ratio (RR) presented with a 95% confidence interval (95% CI). We will test is there is a difference in the risk of having antibiotics prescribed at the index consultation between the two groups.

The PI, who is blinded to group allocation, will perform the statistical analyses in Stata Version 17 according to the SAP. The PI and coauthors remain blinded until after the analyses have been performed, and conclusions are drawn.

### Interim analyses {21b}

No interim analysis is planned.

### Methods for additional analyses (e.g. subgroup analyses) {20b}

We will conduct subgroup analyses of the primary outcome of participants with chronic obstructive pulmonary disease (COPD), comorbid pulmonary disease in general, with a CRP concentration > 50 mg/L or aged ≥ 80 years to determine the risk of effect modification.

### Methods in analysis to handle protocol non-adherence and any statistical methods to handle missing data {20c}

Analyses will be performed as intention to treat (ITT) (pragmatic trial). The primary analysis population will comprise all participants, irrespective of follow-up.

Data will be examined for missing values and outliers. Measures of central tendency and dispersion for continuous study parameters will be portrayed. Extreme or unexpected values will be examined individually for authenticity and data discrepancies addressed where appropriate.

In the case of missing data on the primary endpoint, we will contact the GP who enrolled the participant to clarify whether antibiotics were prescribed at the index consultation. If clarification is not obtained, we will consider it as if antibiotics were prescribed at the index consultation. We expect that the use of an e-CRF for GPs to complete at the time of index consultation will keep missing data on the primary outcome at a minimum. Only observed data will be included in the secondary analyses.

### Plans to give access to the full protocol, participant-level data, and statistical code {31c}

The full protocol, statistical code, and e-CRF templates designed for the study will be available upon request once the report has been published. Participant-level data will not be available.

## Oversight and monitoring

### Composition of the coordinating centre and trial steering committee {5d}

The PLUS-FLUS Trial is coordinated at CAM AAU. MD, Julie Jepsen Strøm, is the PI of this investigator-initiated trial; leads the study design; and is responsible for the project implementation, management, day-to-day support of the trial and for overseeing the data collection. The PI will be supported by the trial steering committee comprising senior researchers at CAM AAU and one independent researcher who will conduct regular meetings to monitor trial progress and provide oversight.

### Composition of the data monitoring committee, its role and reporting structure {21a}

No serious side effects, risks, or disadvantages of applying FLUS have been discovered, and FLUS applies no radioactive radiation, waiving the need for a data monitoring committee.

### Adverse event reporting and harms {22}

There may be side effects, risks, or disadvantages associated with applying FLUS that we do not yet know about, and we ask participating GPs to report any unintended events. All unintended events will be reported to a safety committee. A senior researcher at CAM AAU (MPH) will head the safety committee, which will also comprise a specialist in respiratory medicine, an experienced GP, and a senior researcher experienced with clinical research in general practice.

### Frequency and plans for auditing trial conduct {23}

Access to the data and study documentation necessary for control purposes is allowed for the purposes of independent regulatory authorities.

### Plans for communicating important protocol amendments to relevant parties (e.g. trial participants, ethical committees) {25}

Any modifications to the protocol that will impact the conduct of the study, such as the study objectives, design, patient population, sample size, study procedures, and significant administrative aspects, including the planned or premature end of the study, will be reported to the Ethics Committee of the North Denmark Region. Additionally, the registration on Clinicaltrials.gov will be updated if any of the abovementioned modifications are made.

### Dissemination plans {31a}

The study results will be published in a peer-reviewed medical journal regardless of the outcomes and conclusion. Moreover, the results will be disseminated at various national and international scientific meetings. This study will be reported in accordance with CONSORT guidelines [42]. Authorship will be granted according to the rules of the International Committee of Medical Journal Editors (ICMJE) [43].

## Discussion

This will be the first trial to assess the effect of adding FLUS to usual care on antibiotic use in adults presenting to general practice with symptoms of acute LRTIs. Frequent (over)use of antibiotics in this population is in part due to diagnostic uncertainties surrounding the nonspecific clinical presentation of patients with acute LRTIs.

One strength of the PLUS-FLUS Trial is that it is a randomized controlled trial of unselected general practice patients. It uses a pragmatic comparator of ‘usual care’, which intends to compare the intervention to actual clinical practice. Participating GPs are aware of recommended clinical guidelines and that there are practical and/or pragmatic reasons for not following recommendations that reflect their daily practice.

Furthermore, we perform a very comprehensive data collection with high-quality data on antibiotic prescribing and daily data on participant outcomes during follow-up.

A limitation of the study is that participants and GPs are not blinded to group allocation or to the primary outcome. This was considered impossible because of registration and reporting of the trial protocol, which we assessed, could not be securely concealed from the participating GPs. However, if GPs tend to be improperly less prone to prescribing antibiotics in the + FLUS group, we anticipate that this will become clear during follow-up and result in more antibiotics being prescribed after index consultation up until day 7 or 28 in this group. Moreover, we expect that the clinical course of participants, which is assessed closely during follow-up, will be affected if the GPs improperly withhold antibiotics in one group.

Due to practical issues, participating GPs must be users of POCUS in general practice before trial commencement, which is a population of approximately 19% of Danish GPs. Due to the lack of a fee for performing POCUS in general practice in Denmark, we expect that the population of GPs using POCUS could differ from the general population of Danish general practitioners in several aspects.

As the baseline prescription rate of antibiotics in Denmark is quite low compared to that in other European countries [44], the potential reduction in antibiotic prescriptions by adding FLUS to usual care is reduced compared to that in a setting with a higher prescription rate.

## Trial status

This study was approved by the Ethics Committee of North Denmark Region Protocol version 4.0, 01.11.2023. The first participant was enrolled on November 3, 2023. Recruitment is expected to be completed on April 1, 2024.

## Data Availability

The REDCap© e-CRFs and surveys will be locked after data entry is completed. The complete study dataset will be exported to a secure server at Aalborg University. The PI and coauthors with a data processing agreement will have unlimited access to the final dataset before publication. The exported dataset will be stored for 10 years and then deleted.

## Abbreviations

LRTI: Lower respiratory tract infections
CAP: Community-acquired pneumonia
GP: General practitioner
FLUS: Focused lung ultrasonography
CAM AAU: Center for General Practice at Aalborg University
WHO: World Health Organization
CRP: C-reactive protein
POCUS: Point-of-care ultrasonography
PI: Primary investigator
FMK: Shared medication records
DSAM: Danish Society of General Practitioners
LUS-OSAUS: Lung Ultrasound-Objective Structured Assessment of Ultrasound Skills
EFSUMB: European Federation of Societies for Ultrasound in Medicine and Biology
e-RCF: Electronic case report form
POCT: Point-of-care test
SNOSE: Sequentially numbered opaque sealed envelope
REDCap©: Research Electronic Data Capture
SMS: Short message services
GDPR: The General Data Protection Regulation
SAP: Statistical analysis plan
COPD: Chronic obstructive pulmonary disease
ITT: Intention to treat
